# From Extracellular Vesicles-Related Genes to Angiogenesis: KRT7 as a Modulator of the VEGF/VEGFR signaling-dependent angiogenesis in Pancreatic Adenocarcinoma

**DOI:** 10.7150/ijms.129654

**Published:** 2026-05-01

**Authors:** Tianyin Ma, Xiangdong Gongye, Cairang Dongzhi, Shuxian Ma, Yibo Chai, Wing-Wa Guo, Qikun Wang, Ming Tian

**Affiliations:** 1 Department of Hepatobiliary & Pancreatic Surgery, Zhongnan Hospital of Wuhan University, Wuhan, 430071, China.; 2 Department of Chemistry and Molecular Biology, Sahlgrenska Akademin, Göteborg Universitet, Gothenburg, Vastra Gotalands, Sweden.; 3 Department of Oncology, The Sixth Hospital of Wuhan, Affiliated Hospital of Jianghan University, Wuhan 430072, Hubei, China.; 4 Department of General Surgery, Pancreatic Disease Center, Ruijin Hospital, Shanghai Jiao Tong University School of Medicine, Shanghai 200025, China.

**Keywords:** KRT7, VEGF/VEGFR signaling-dependent angiogenesis, pancreatic adenocarcinoma, prognostic model, extracellular vesicles

## Abstract

**Background & Aims**: Pancreatic adenocarcinoma (PAAD) is a highly aggressive malignancy with limited treatment options and a poor prognosis. Extracellular vesicles (EVs), which play a central role in intercellular communication, have emerged as promising non-invasive biomarkers for both diagnosis and prognosis. This study aimed to identify EV-related genes, construct a more accurate prognostic model using data from multiple databases, and explore the functional roles of key genes in PAAD.

**Methods & Results**: Four publicly available datasets, PAAD_ExoRbase, TcgaTargetGtex-PAAD, GSE62452_GPL6244, and GSE78229_GPL6244, were analyzed, leading to the identification of 40 differentially expressed EV-related genes. Using the Least Absolute Shrinkage and Selection Operator (LASSO) Cox regression, five prognostic genes, *KRT7*, *KRT19*, *CXCL5*, *TNFRSF13C*, and *GP1BA*, were selected to construct a nomogram model for predicting patient outcomes. Single-cell analysis showed that these five genes were expressed in T-proliferative, malignant, and ductal cells. Analyses of immune cell infiltration and immune checkpoints further supported the model's prognostic performance. The regulatory role of the key gene *KRT7* was validated in an in-house cohort and confirmed through in vitro experiments and RNA sequencing. Furthermore, analyses of conditioned medium, EV isolation and characterization, and endothelial tube formation assays demonstrated that EVs derived from CFPAC-1 cells carrying *KRT7* enhanced PAAD angiogenesis through VEGF/VEGFR signaling.

**Conclusion:**

A robust prognostic model was established that connects overall survival with immune status in patients with PAAD. This model may offer valuable guidance for tailoring personalized therapies according to patient subtypes. The mechanisms underlying the regulatory role of the key gene *KRT7* in this model were further investigated, revealing *KRT7* as a pivotal regulator of VEGF/VEGFR signaling-mediated angiogenesis in PAAD.

## Introduction

Pancreatic adenocarcinoma (PAAD) is among the most lethal malignancies, with a five-year survival rate of only 10% 1. More than 80% of patients are diagnosed at advanced or metastatic stages, largely because the disease remains asymptomatic in its early phases 2. Despite improvements in surgical approaches, effective treatments for advanced PAAD are still lacking, and patient outcomes remain poor. Although *KRAS*, *CDKN2A*, *SMAD4*, and *TP53* have been identified as key driver genes in PAAD, progress in developing targeted therapies has been limited 3. Further, patients frequently develop rapid resistance to conventional chemotherapy, underscoring the need for a deeper understanding of the tumor microenvironment, immune landscape, and mechanisms of intercellular communication 4,5.

Extracellular vesicles (EVs) have attracted attention as promising biomarkers due to their ability to transport biomolecules, including proteins, lipids, and nucleic acids, with high stability and abundance in biological fluids 6. EVs contribute to tumor initiation, progression, and metastasis by facilitating intercellular communication. Evidence suggests that the packaging of biomolecules into EVs is a regulated rather than a random process. For example, EVs derived from breast carcinoma and prostate adenocarcinoma have been shown to promote tumor progression through the transfer of specific microRNAs (miRNAs) 7. Similarly, exosomal miR-200 from metastatic breast cancer enhances epithelial-mesenchymal transition (EMT) and promotes metastasis in less aggressive breast cancer cells 8.

In this study, RNA expression data associated with PAAD-derived EVs were analyzed using datasets from ExoRbase, UCSC Xena, and GEO. Single-cell analysis was performed to trace the cellular origin of these EVs. The role of *KRT7* and its underlying mechanisms in PAAD was investigated. This work aimed to develop a robust prognostic model for PAAD and to provide insights into potential biomarkers and the molecular mechanisms driving this aggressive disease.

## Materials and Methods

### Dataset preparation and preprocessing

Information on blood-circulating EV-associated RNA expression profiles from 164 patients with PAAD and 118 healthy donors was obtained from the ExoRbase database. These data were integrated and log-transformed using log2(TPM + 1) to generate the PAAD_ExoRbase dataset.

RNA expression profiles and corresponding clinical data for tumor and normal samples were retrieved from Xenabrowser_TcgaTargetGtex (UCSC Xena). This dataset included 178 tumor samples, 4 peritumoral samples, and 167 GTEx samples for normal-tumor comparisons. Expression values were log2-transformed as log2(TPM + 0.001). Tumor samples lacking clinical information or with an overall survival (OS) time of zero were excluded, leaving 177 PAAD samples, referred to as the TCGA-PAAD dataset. The associated clinical characteristics are summarized in **Table S1**.

RNA expression matrix files GSE62452_GPL6244 and GSE78229_GPL6244 were downloaded from the GEO database, along with their corresponding probe annotation file (GPL6244). Probes were mapped to gene symbols, and those corresponding to multiple genes were excluded. When multiple probes corresponded to a single gene, median expression values were used. Duplicate and incomplete samples were removed before analysis. Clinical information for these datasets is provided in **Tables S2**,** S3**, and **S4**.

### Differential expression analysis

Differentially expressed genes (DEGs) were identified using the “limma” R package, with criteria of | log_2_(fold change, FC) | > 1 and adjusted *p* < 0.05. Heatmaps and volcano plots were generated using the “ComplexHeatmap” and “ggplot2” packages, respectively. The intersecting DEGs from the PAAD_ExoRbase and TcgaTargetGtex-PAAD datasets were selected for further analyses.

### Enrichment analysis

Gene ontology (GO) analysis was conducted to annotate the molecular functions (MFs), cellular components (CCs), and biological processes (BPs) of the genes. Kyoto Encyclopedia of Genes and Genomes (KEGG) pathway analysis was also performed to explore potential functional roles. Both analyses were conducted using the “ClusterProfiler” package, with *p* < 0.05 considered statistically significant.

### Mutation analysis

Gene mutations were analyzed using data from multiple sources, including 177 tumor tissue samples from the TCGA-PAAD dataset, 178 samples from the TCGA-PAAD single-nucleotide variation (SNV) cohort, and 184 samples from the TCGA-PAAD copy number variation (CNV) cohort. Overlapping samples across these datasets were selected for detailed analysis, yielding 170 samples for SNV analysis and 176 samples for CNV analysis.

### Construction and validation of the prognostic signature

Univariate Cox regression analysis (UVA) was performed on the overlapping differentially expressed genes (DEGs) to identify EV-related survival genes, using a significance threshold of *p* < 0.05. Subsequently, a least absolute shrinkage and selection operator (LASSO) Cox regression analysis was performed to identify redundant genes and develop a prognostic signature.

The following formula was employed for calculating the risk score (RS) for individual patients:



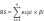



Where *n* represents the number of genes, *expi* is the expression level of gene i, and *βi* is the corresponding regression coefficient.

Patients were stratified into high-risk (HRG) and low-risk (LRG) groups according to the median risk score (RS). Overall survival (OS) between the two groups was compared using the “survminer” package, while time-dependent receiver operating characteristic (ROC) curves were generated with the “timeROC” package to assess the predictive performance of the model. Univariate and multivariate Cox regression analyses (UVA and MVA) were further conducted to evaluate whether the RS served as an independent prognostic factor. The same formula was then applied to the validation datasets (GSE62452_GPL6244 and GSE78229_GPL6244) to calculate RS and validate the prognostic model.

### Single-cell analysis

The GSE111672 dataset was downloaded from the GEO database. Single-cell data from GSE111672 were analyzed to identify the potential cellular origins of the five prognostic genes, and the RS of cell subpopulations were calculated using the prognostic model.

### Estimation of immune cell infiltration in the tumor immune microenvironment (TIME)

The relative abundance of immune cell infiltration in the TIME was quantified using the single-sample gene set enrichment analysis (ssGSEA) algorithm. Gene sets corresponding to each TIME-infiltrating immune cell subtype, including macrophages, regulatory T cells, activated dendritic cells (DCs), activated CD8^+^ T cells, and natural killer (NK) cells, were obtained from Charoentong *et al*. 9 research. Enrichment scores from ssGSEA were used to represent the relative abundance of each immune cell subtype in each sample.

### In-house cohort

The in-house cohort included 33 patients who underwent surgical resection at the Department of Hepatobiliary and Pancreatic Surgery, Zhongnan Hospital of Wuhan University, between 2020 and 2023. None of the patients received neoadjuvant chemotherapy. Written informed consent was obtained from all participants before enrollment, in accordance with the Declaration of Helsinki. The study was approved by the Medical Ethics Committee of Zhongnan Hospital of Wuhan University (2025004K). All sample collection and processing procedures were carried out in strict adherence to ethical standards. Clinical details of the in-house cohort are summarized in **Table S5**.

### Cell culture and transfection

The CFPAC-1 PAAD cell line was obtained from Procell Life Science & Technology Co., Ltd. and maintained in Iscove's Modified Dulbecco's Medium (IMDM) supplemented with 10% fetal bovine serum (FBS) and 1% penicillin-streptomycin (P/S). Human umbilical vein endothelial cells (HUVECs), also purchased from Procell Life Science & Technology Co., Ltd., were cultured in Dulbecco's Modified Eagle Medium (DMEM) containing 10% FBS and 1% P/S. Transfection experiments were carried out according to previously published methods 10,11.

### RT-qPCR, immunohistochemistry (IHC), and western blotting

RT-qPCR, IHC, and Western blotting were performed according to protocols detailed in our previously published studies 12,13. The primer sequences used for RT-qPCR are listed in **Table S6**, and details of the antibodies used for IHC and Western blotting are provided in **Table S7**.

### *In vitro* experiments

*In vitro* experiments, including colony formation, wound-healing, and Tanswell assays, were conducted according to protocols described in earlier research 14. All techniques adhered to previously validated methods.

### RNA sequencing (RNA-seq)

RNA-seq was performed according to the experimental procedures outlined in the previous literature 15. The raw sequencing data have been deposited in the GEO database (GSE283773) for reference and future research.

### Endothelial tube formation assays

Tube formation assays were conducted as previously described 16. Briefly, conditioned media were collected from CFPAC-1 cells transfected with siKRT7 or negative control siRNA (NC). For specific treatments, VEGF (HY-P7110A, MCE, USA) or Axitinib (HY-10065, MCE) was added to the conditioned media. HUVECs were seeded in Matrigel-coated wells and cultured with the prepared conditioned media for 6-8 hours, after which tube formation was evaluated and imaged using an inverted fluorescence microscope (IX73, Olympus, Japan). Quantitative analysis of angiogenesis-related parameters, including the area covered, total tube length, total number of branching points, and total number of loops, was performed using WimTub (https://www.wimasis.com).

### Enzyme-linked immunosorbent assay (ELISA)

VEGF levels in the cell culture supernatant were determined using a Human VEGF ELISA Kit (KHG0111, Thermo Fisher Scientific) according to the manufacturer's protocol. Briefly, harvested cell culture supernatants were added to the precoated enzyme plate, and absorbance was measured with a microplate reader after the addition of stop solution.

### Isolation of EVs

CFPAC-1 cells were cultured in medium supplemented with EV-depleted serum (XP Biomed), and the medium was collected for EV isolation. EVs were isolated by differential ultracentrifugation. Briefly, the collected medium was centrifuged at 300 × g for 10 min and 2,000 × g for 20 min to remove cells and cellular debris, followed by centrifugation at 10,000 × g for 30 min to remove larger vesicles. The supernatant was then filtered through a 0.22 μm filter and ultracentrifuged at 120,000 × g for 70 min at 4 °C. The pellet was resuspended in saline and centrifuged again at 120,000 × g for 70 min for further purification. The final EV pellet was resuspended in saline and stored at -80 °C until further use. All centrifugation procedures were performed at 4 °C.

### Resistive pulse sensing (RPS)

To assess the size distribution of EVs, resistive pulse sensing (RPS) analysis was performed using a NanoCoulter counter (Resun Technology Co., Ltd., Shenzhen, China) based on the Coulter principle. A customized chip was used according to the specific measurement range required for analyte detection.

### Transmission electron microscopy (TEM)

TEM was used to characterize the morphology of EVs. Purified EVs suspended in PBS were placed onto copper grids, stained with 2% uranyl acetate, and dried under an infrared lamp. The grids were then examined using a transmission electron microscope (Talos L120C G2, Thermo Fisher Scientific, USA).

### Immunoelectron microscopy

Immunoelectron microscopy was used to detect *KRT7* on EVs. For immunogold labeling, EVs in saline at a concentration of 1 × 10^9^ particles/mL were loaded onto glow-discharged copper grids, followed by blocking and incubation with a rabbit anti-human polyclonal antibody against *KRT7* (Proteintech, 17513-1-AP). The grids were then incubated with a 10 nm gold-conjugated anti-rabbit secondary antibody (Solarbio, K1034G-G35). After each incubation step, the grids were washed five times with saline and ten times with ultrapure water. The samples were subsequently stained with 2% uranyl acetate and observed under a transmission electron microscope (Talos L120C G2, Thermo Fisher Scientific, USA).

### Direct treatment with EVs

After isolation, EVs were directly added to endothelial cell culture medium for subsequent functional assays. Briefly, 100 μL of EV suspension, diluted according to the particle concentration determined by RPS, was added to each well. Referring to our previously published study, the final concentration of EVs in the culture medium was set at 1 × 10^10^ particles/mL 17. Freshly isolated EVs from the same preparation batch were used in each assay to ensure experimental consistency.

### Statistical analysis

Statistical analyses were conducted using R software V4.1.2. The Wilcoxon test was used for comparisons between two groups, and the Kruskal-Wallis test for comparisons across multiple groups. Survival analyses were conducted using the Kaplan-Meier (KM) method, with the log-rank test used to assess differences in OS between groups. UVA and MVA were employed to determine the independent prognostic value of RS when combined with clinical features. ROC curves were generated to evaluate the predictive efficiency of the risk model for 1-, 3-, and 5-year OS. Statistical significance was defined as *p* < 0.05. By convention: **** indicates *p* < 0.0001, *** indicates *p* < 0.001, ** indicates *p* < 0.01, indicates *p* < 0.05, and n.s. indicates non-significance.

## Results

### Differential expression and UVA of EV-related genes

**Figure 1:**
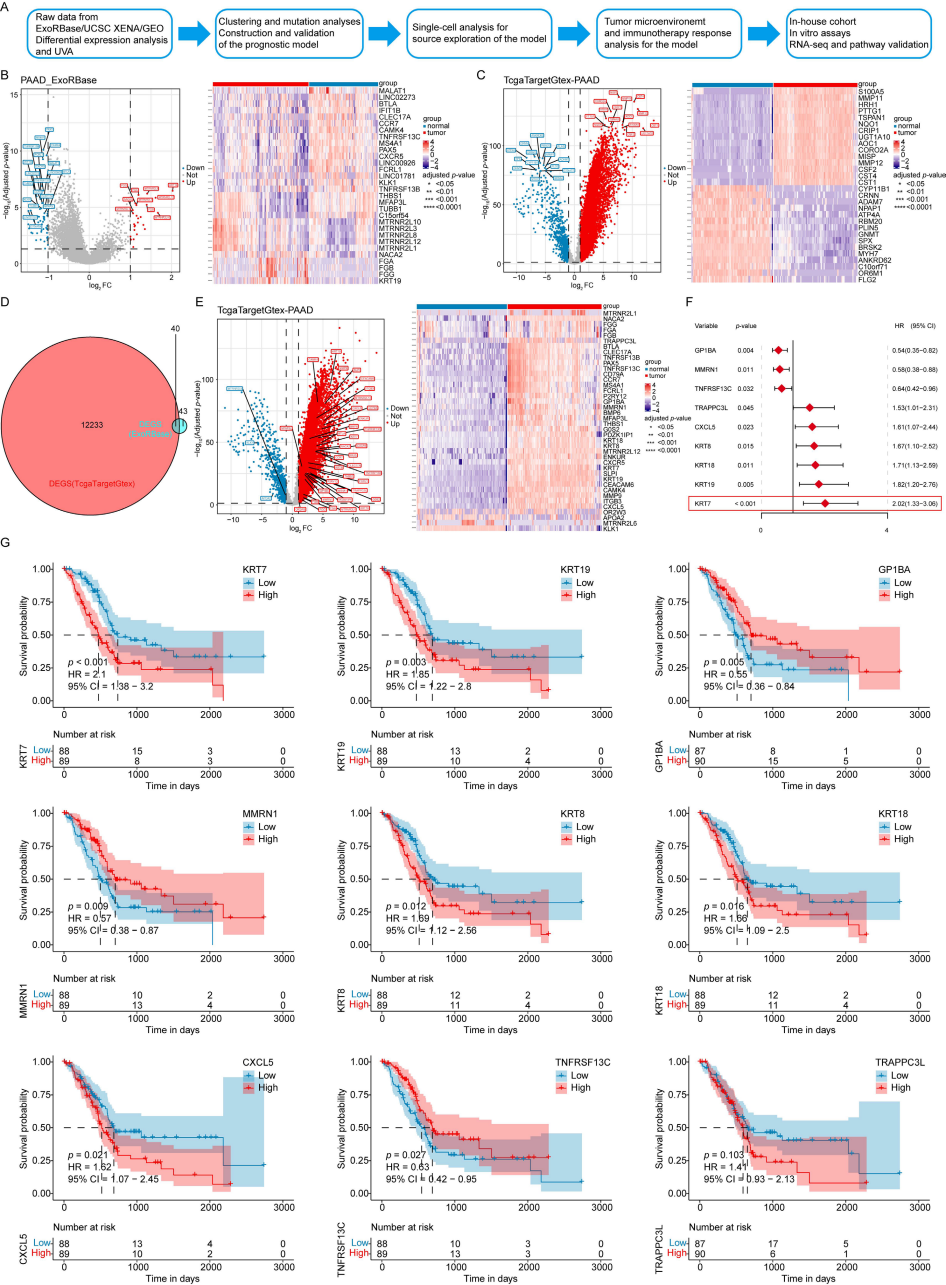
** Differential expression and UVA of EV-related genes.** (**A**) Workflow of the data analysis process. (**B**) Volcano plot and heatmap of differentially expressed genes in the PAAD_ExoRbase cohort. (**C**) Volcano plot and heatmap of differentially expressed genes in the TcgaTargetGtex-PAAD cohort. (**D**) Overlapping exogenes identified in PAAD. (**E**) Volcano plot and heatmap of 40 differentially expressed EV-related genes in the TcgaTargetGtex-PAAD cohort. (**F**) UVA identifying 9 genes associated with OS, with *KRT7* showing the highest HR. (**G**) KM survival curves. ****p ≤ 0.0001; ***p ≤ 0.001; **p ≤ 0.01; *p ≤ 0.05; n.s., no significant.

Initially, DEGs were analyzed in the PAAD EVs (PAAD_ExoRbase) cohort (**Figure 1A**). Using a filtering threshold of |log₂ FC| > 1 and adjusted *p* < 0.05, 83 DEGs were identified, including 25 upregulated genes and 58 downregulated genes. The expression levels of *KRT19, MTRNR2L12, MTRNR2L8*, and *FGG,* among other genes, were significantly upregulated, while genes such as *TNFRSF13B, PAX5*, and *CCR7,* among other genes, showed downregulation (**Figure 1B**). Next, the TCGA Target GTEx-PAAD cohort was analyzed using the same filtering criteria, yielding 12,273 DEGs: 11,510 upregulated and 763 downregulated. Among these, *MISP, CST4, MMP12*, *CORO2A,* and some other genes showed significant upregulation, whereas *CRNN, ANKRD62, ATP4A*, *RBM20,* and other genes were significantly downregulated (**Figure 1C**). By intersecting the DEGs from the two cohorts, 40 EV-related genes were identified as overlapping. Within the TcgaTargetGtex-PAAD cohort, genes such as *KRT19, MMP9, SLPI*, and *CEACAM6*, among other genes, were significantly upregulated, while *MTRNR2L6, KLK1*, *APOA2*, and some other genes were significantly downregulated (**Figure 1D-E**).

To explore the association between these 40 EV-associated DEGs and the prognosis of PAAD patients, UVA was performed using the TCGA-PAAD dataset. Nine EV survival-related genes were found: *KRT7, GP1BA, KRT19, MMRN1, KRT18, KRT8, CXCL5, TNFRSF13C*, and *TRAPPC3L*. Among these, six genes, *KRT7, KRT19, KRT18, KRT8, CXCL5,* and *TRAPPC3L* (hazard ratio [HR] > 1) were identified as risk factors, while three genes, *GP1BA, MMRN1,* and *TNFRSF13C* (HR < 1) were protective factors for PAAD patients (**Figure 1F**).

The prognostic significance of these genes was further evaluated by dividing patient data into high- and low-expression groups. The KM curves showed that high expression levels of *KRT7*, *KRT19*, *KRT18*, *KRT8*, and *CXCL5* were associated with lower OS. In comparison, high expression levels of *GP1BA*, *MMRN1*, and *TNFRSF13C* were associated with higher OS rates, consistent with the UVA results (**Figure 1G**).

### Clustering and mutation analyses of the survival-associated genes

**Figure 2:**
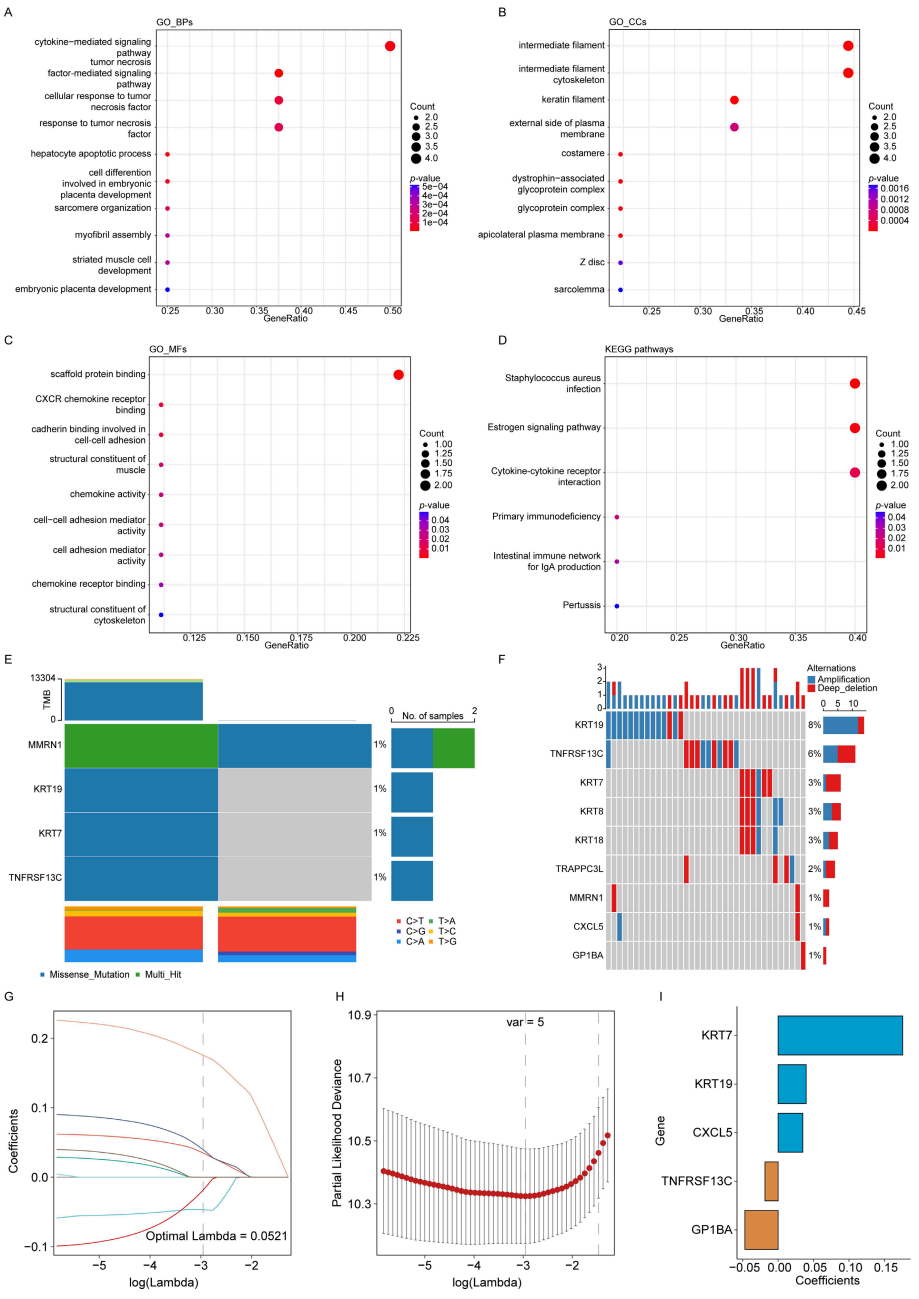
** Clustering and mutation analyses of prognostic genes and construction of the model.** (**A**) GO biological processes (BPs). (**B**) GO cellular components (CCs). (**C**) GO molecular functions (MFs). (**D**) KEGG enrichment analysis. (**E**) SNV analysis of EV survival-related genes. (**F**) CNV analysis of EV survival-related genes. (**G**) Trajectories of 9 independent variables analyzed using LASSO regression. (**H**) Confidence intervals for each lambda value. (**I**) LASSO coefficient profiles of the 5 key prognostic genes.

The biological roles of the nine EV-related survival genes were further explored through enrichment analyses. GO analysis showed that these genes were involved in BPs, including cytokine- and tumor necrosis factor-mediated signaling (**Figure 2A**). In terms of CCs, they were primarily associated with the intermediate filament and the intermediate filament cytoskeleton (**Figure 2B**). For MFs, the genes were mainly enriched in scaffold protein binding and CXCR chemokine receptor binding (**Figure 2C**). KEGG pathway analysis further indicated significant enrichment in pathways including *Staphylococcus aureus* infection and estrogen signaling (**Figure 2D**).

To extend the analysis, proteomic data for PAAD at Levels 3 and 4 were obtained from the TCPA database. However, no corresponding data for the EV-related survival genes were available in the TCPA dataset, which prevented further investigation at the proteomic level.

Mutations in the EV survival-related genes associated with PAAD were also investigated. **Figure 2E** illustrates SNVs within the genes, showing relatively few SNVs among the EV survival-related genes. Only two samples showed SNVs, with four gene mutations (*MMRN1*, *KRT19*, *KRT7*, and *TNFRSF13C*) detected at a frequency of 1%. **Figure 2F** presents the CNV data, highlighting relatively high mutation rates for *KRT19* (8%) and *TNFRSF13C* (6%).

### Construction and validation of the prognostic model

**Figure 3:**
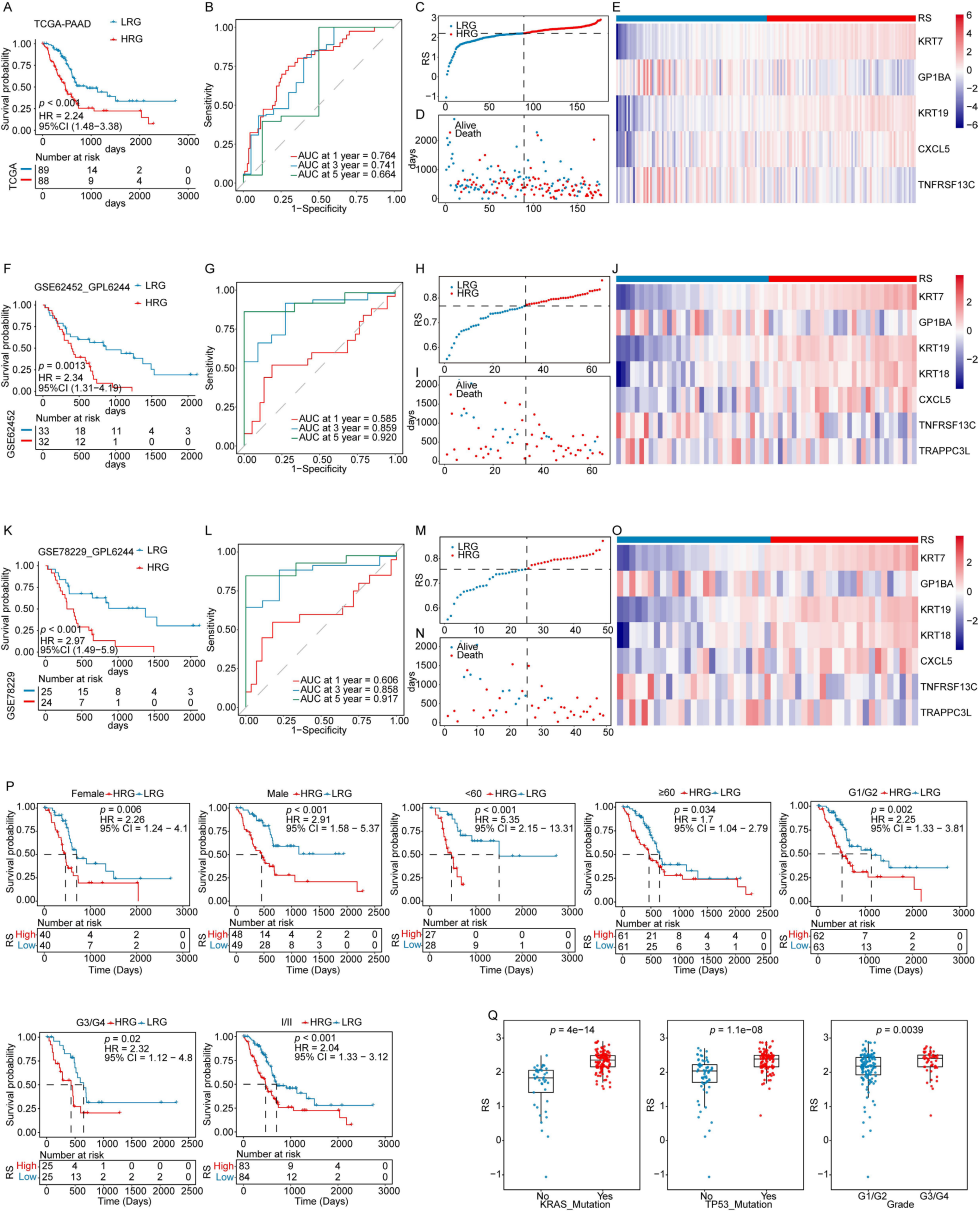
** Validation of the prognostic model.** (i) Validation in the TCGA-PAAD training cohort: (**A**) KM survival curves for high-risk group (HRG) and low-risk group (LRG). (**B**) Time-dependent ROC curves. (**C**) Risk score (RS) distribution for each sample. (**D**) Survival time distribution for each sample. (**E**) Heatmap displaying the expression levels of prognostic genes in both HRG and LRG. (ii) Validation in the GSE62452_GPL6244 validation cohort: (**F**) KM survival curves for HRG and LRG. (**G**) Time-dependent ROC curves. (**H**) RS distribution for each sample. (**I**) Survival time distribution for each sample. (**J**) Heatmap of prognostic gene expression in HRG and LRG. (iii) Validation in the GSE78229_GPL6244 validation cohort: (**K**) KM survival curves for HRG and LRG. (**L**) Time-dependent ROC curves. (**M**) RS distribution for each sample. (**N**) Survival time distribution for each sample. (**O**) Heatmap displaying the expression levels of prognostic genes in both HRG and LRG. (**P**) KM analysis of subgroups with different clinical features. (**Q**) Boxplots showing the association between RS and clinical features.

The impact of the nine genes on OS was analyzed using LASSO Cox regression, yielding five selected genes and their corresponding coefficients. These coefficients were used to calculate the RS for each patient (**Figure 2G-I**). The impact of the RS on OS, constructed from the five selected genes, was evaluated. In the TCGA-PAAD dataset, patients were classified as LRG or HRG based on the median RS. KM analysis revealed that patients in the HRG had significantly shorter OS times than those in the LRG (**Figure 3A**). The prognostic model demonstrated good predictive performance for OS in the TCGA-PAAD training cohort, with areas under the curve (AUC) of 0.764, 0.741, and 0.664 for 1-, 3-, and 5-year survival, respectively (**Figure 3B**). Furthermore, the RS distribution was continuous, with no outliers or extreme values detected, and a clear association between lower RS and longer OS was observed (**Figure 3C-D**).

The robustness of the prognostic model developed using the TCGA-PAAD training cohort was further evaluated in two independent datasets (GSE62452_GPL6244 and GSE78229_GPL6244). Applying the same algorithm, risk scores (RS) were calculated for each sample in the validation cohorts. Kaplan-Meier analyses, ROC curves, and the distributions of RS and survival time consistently demonstrated that patients in the HRG had significantly shorter OS than those in the LRG. The model exhibited strong predictive performance for OS across both validation datasets (**Figure 3F-I, 3K-N**). The expression levels of key prognostic genes were assessed in both the training and validation cohorts, revealing consistent patterns across datasets (**Figure 3E, 3J, 3O**). These results confirm that the prognostic model is robust and reliable, offering an effective tool for predicting outcomes in patients with PAAD.

### RS as an independent prognostic factor (IPF)

The potential of the RS as an IPF was assessed by stratifying patients in the TCGA-PAAD cohort according to clinical characteristics. OS analyses within these subgroups showed that patients in the HRG had poorer outcomes than those in the LRG across most clinical categories, supporting the robustness of the prognostic model (**Figure 3P**). Analysis of the relationship between RS and clinical features revealed significant differences in RS across tumor grade, KRAS mutation status, and TP53 mutation status (**Figure 3Q, Fig. S1**).

**Figure 4:**
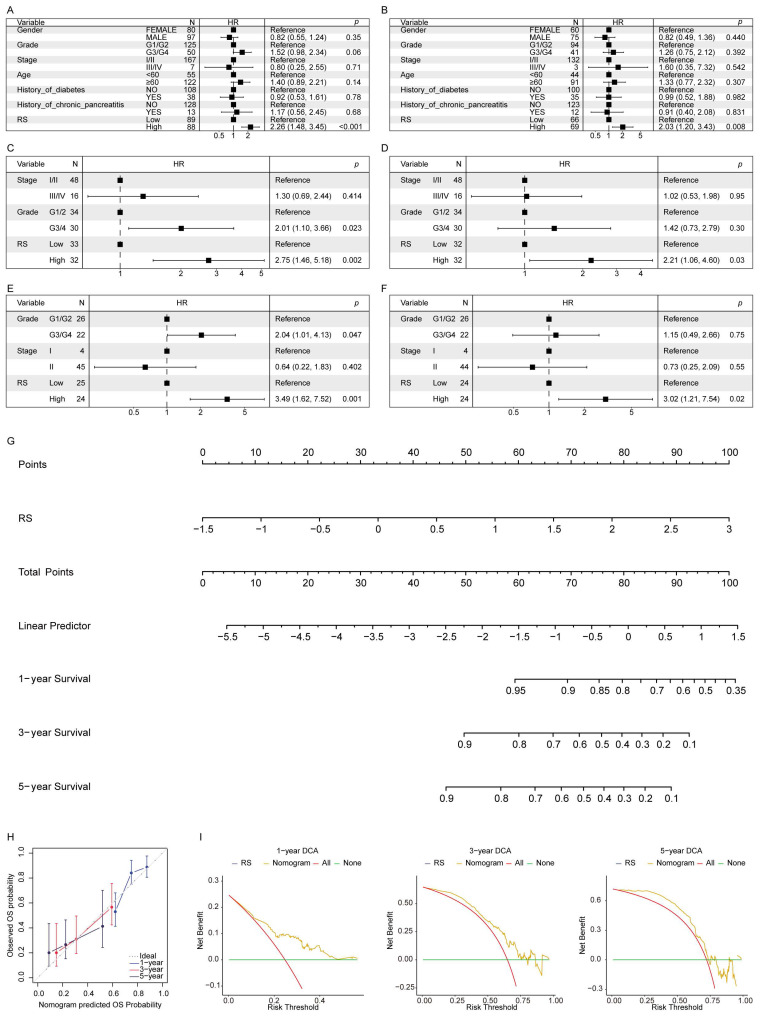
** The risk score as an independent prognostic indicator.** (**A**) UVA in the TCGA-PAAD cohort. (**B**) MVA in the TCGA-PAAD cohort. (**C**) UVA in the GSE62452_GPL6244 validation cohort. (**D**) MVA in the GSE62452_GPL6244 validation cohort. (**E**) UVA in the GSE78229_GPL6244 validation cohort. (**F**) MVA in the GSE78229_GPL6244 validation cohort. (**G**) The nomogram model constructed using the TCGA-PAAD cohort. (**H**) Calibration curves for 1-, 3-, and 5-year OS in the nomogram model. (**I**) DCA curves for 1-, 3-, and 5-year survival based on the clinical model.

UVA demonstrated a significant association between RS and OS in the TCGA-PAAD cohort (**Figure 4A**). After adjusting for potential confounders, MVA confirmed that RS remained an independent predictor of OS (**Figure 4B**). Similar findings were observed in the two validation cohorts, further supporting the reliability of RS as an IPF (**Figure 4C-F**).

A nomogram was constructed to develop a clinically applicable predictive model for PAAD patients using the RS and other IPFs to estimate OS at 1-, 3-, and 5-years (**Figure 4G**). The calibration curve demonstrated good agreement between predicted and observed outcomes, showing the nomogram's predictive accuracy (**Figure 4H**). Decision curve analysis (DCA) further validated the model's clinical utility, suggesting its applicability across a wide range of clinical scenarios (**Figure 4I**).

### Single-cell analysis of the signature source and cell subpopulation RS

**Figure 5:**
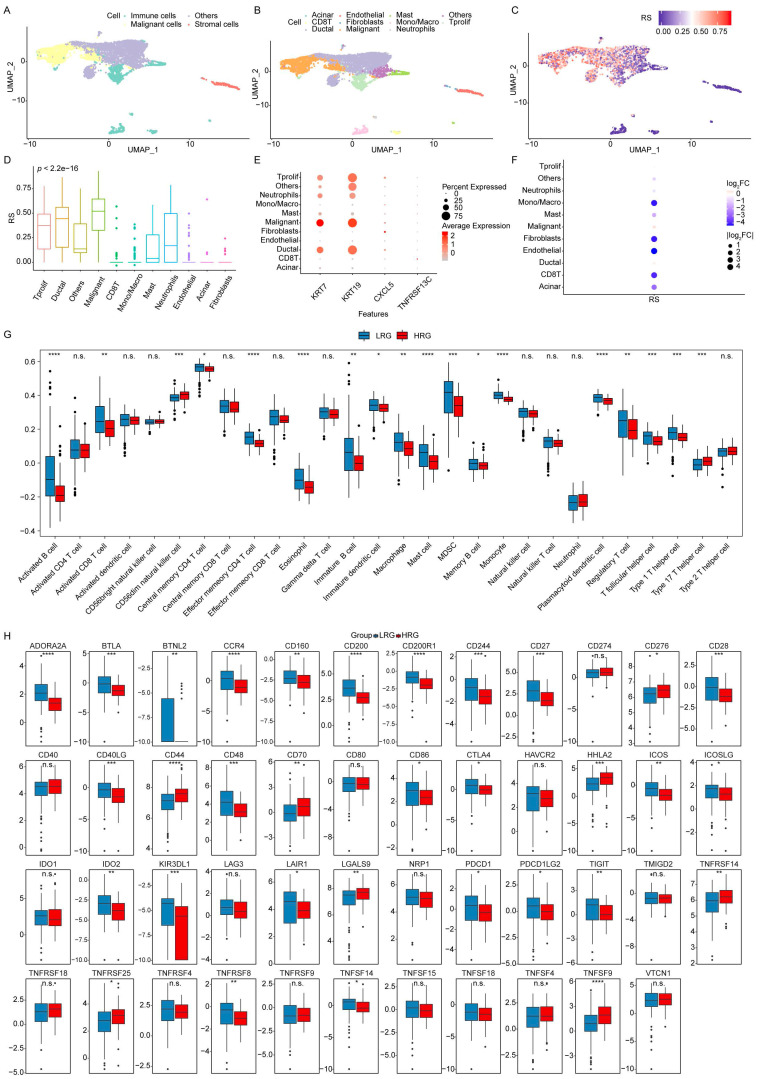
** Single-cell and ssGSEA analysis of the prognostic model.** (**A**) Distribution of malignant, stromal, and immune cells. (**B**) Distribution of individual cell subpopulations. (**C**) Enrichment of RS in different cell subpopulations. (**D**) Distribution of RS across cell subpopulations. (**E**) Expression of prognostic genes in individual cell subsets. (**F**) Log_2_ FC in RS across cell subsets. (**G**) Comparison of immune cell infiltration in the TIME between HRG and LRG using ssGSEA. (**H**) Comparison of the expression profiles of immune checkpoint between HRG and LRG. ****p ≤ 0.0001; ***p ≤ 0.001; **p ≤ 0.01; *p ≤ 0.05; n.s., non-significant.

Single-cell analysis was performed to trace the cellular origin of the five selected EV-related survival genes and to assess RS distribution across cell subpopulations. Cells from the GSE111672 dataset were clustered, and the RS distribution was visualized (**Figure 5A-C**). Differential analysis showed that T-proliferative cells, ductal cells, and malignant cells exhibited higher RS values (**Figure 5D**). Analysis of gene expression across cell subsets revealed that KRT7 and KRT19, identified as high-risk genes, were major contributors to these elevated scores (**Figure 5E**).

Furthermore, log₂ fold-change analysis of RC across cell subsets indicated that *CXCL5* and *TNFRSF13C*, classified as low-risk genes, were enriched in Mono/Macro cells, fibroblasts, and endothelial cells. These results suggest that *CXCL5* may play an immunosuppressive role through Mono/Macro cells, influencing the tumor immune microenvironment (TIME) (**Figure 5F**). *KRT7* and *KRT19* appear to serve as key markers of malignant cells in PAAD, whereas *CXCL5* and *TNFRSF13C* may indirectly promote tumor progression by modulating the TIME through immune cell regulation. These results highlight the value of the RS model in understanding and predicting PAAD progression.

### Analysis of the RS and TIME

The association between the RS and immune status was further investigated by comparing immune cell infiltration within the TIME between the HRG and LRG groups, using boxplots generated from ssGSEA analysis. The results showed that CD56^dim NK cells and Type 17 helper T cells exhibited higher infiltration levels in the HRG. In comparison, the LRG demonstrated significantly greater infiltration of Activated B cells, Effector memory CD4 T cells, Eosinophils, Mast cells, Monocytes, and Plasmacytoid DCs (**Figure 5G**).

Immune checkpoints, key regulators of immune activation, were analyzed for differential expression between the HRG and LRG. Among the 47 immune checkpoints assessed, 32 showed significant differences, indicating distinct immune regulatory landscapes between the two groups (**Figure 5H**).

### Validation of the key factor *KRT7* in clinical cohort and *in vitro* assays

**Figure 6:**
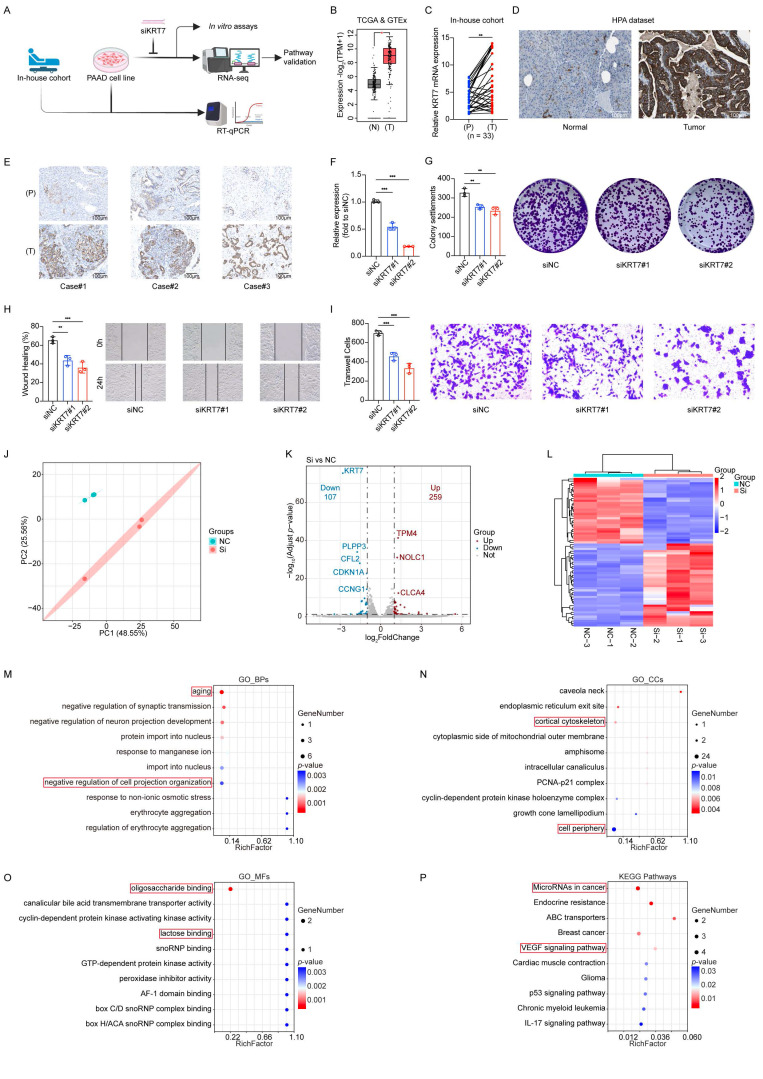
** Validation of the key factor *KRT7* in clinical cohorts and *in vitro* assays.** (**A**) Workflow for clinical cohort and *in vitro* assay validation. (**B**) Validation using TCGA & GTEx data. (**C**) Validation in the in-house cohort. T: Tumor, P: Paracancerous tissue. (**D**) Validation using the HPA dataset. (**E**) Representative IHC images. (**F**) RT-qPCR results showing *KRT7* knockdown efficiency in the CFPAC-1 cell line. (**G**) Colony formation assay results and corresponding statistical graph. (**H**) Wound healing assay results and statistical graph. (**I**) Transwell Matrigel invasion assay results and statistical graph. (**J**) Principal component analysis (PCA) component determination. (**K**) Volcano plot of differential analysis. (**L**) Heatmap of differential analysis. (**M**) GO BPs analysis. (**N**) GO CCs analysis. (**O**) GO MFs analysis. (**P**) KEGG enrichment analysis. Data are expressed as mean ± SD from three independent experiments. ****p ≤ 0.0001; ***p ≤ 0.001; **p ≤ 0.01; *p ≤ 0.05; n.s., no significant.

*KRT7* encodes a keratin family protein specifically expressed in the vasculature and ducts of glandular tissues, playing a role in cytoskeletal formation and in the regulation of cell motility. Identified as a high-risk gene by the prognostic model and single-cell analysis, *KRT7* was further validated in an internal cohort, and its function was explored in the PAAD cell line (**Figure 6A**). The results from TCGA & GTEx datasets, and the in-house cohort, demonstrated significantly elevated mRNA expression of *KRT7* in tumors compared to paracancerous tissues (**Figure 6B-C**). Data from the Human Protein Atlas (HPA) and paired tumor and adjacent normal tissues from three patients confirmed higher *KRT7* protein levels in tumors via IHC (**Figure 6D-E**).

*In vitro* experiments established a *KRT7* knockdown model using the CFPAC-1 cell line (**Figure 6F**). Functional assays showed that *KRT7* knockdown inhibited cell proliferation, migration, and invasion in PAAD cells (**Figure 6G-I**).

RNA-seq and enrichment analyses revealed functional changes associated with *KRT7* knockdown (**Figure 6J-L, Figure S2**). In BPs, enrichment of terms such as "aging" suggested that *KRT7* knockdown increases cellular stress accumulation, functional decline, or markers of senescence, potentially suppressing tumor cell proliferation and division. Enrichment of "negative regulation of cell projection organization" underscored *KRT7*'s role in cytoskeletal organization and in the formation of membrane protrusions. These findings indicate that *KRT7* knockdown disrupts cell polarity and motility, reducing migration and invasion ability (**Figure 6M**).

In CCs, terms such as "cortical cytoskeleton" and "cell periphery" highlighted *KRT7*'s role in maintaining cytoskeletal structure and cellular boundaries. These results confirm that *KRT7* functions as a key cytoskeletal component, with its knockdown impairing cell polarity, adhesion, and migration (**Figure 6N**).

In MFs, terms like "oligosaccharide binding" and "lactose binding" suggested that *KRT7* knockdown might disrupt interactions with glycan-related molecules, including cell adhesion molecules and extracellular matrix proteins, which further explain the observed reduction in invasion capacity (**Figure 6O**).

KEGG enrichment analysis revealed significant enrichment in pathways such as “MicroRNAs in cancer,” suggesting that *KRT7* knockdown may influence PAAD cell invasion and migration by regulating cancer-associated microRNAs. Enrichment of the “VEGF signaling pathway” indicated that *KRT7* knockdown could inhibit tumor angiogenesis, limiting invasion, metabolic adaptation, and nutrient supply in PAAD (**Figure 6P**).

These results indicate that *KRT7*, as a high-risk gene in PAAD, promotes cancer cell proliferation, migration, and invasion by preserving cytoskeletal structure and cellular polarity. *KRT7* may enhance tumor angiogenesis, metabolic adaptation, and immune evasion by regulating key oncogenic pathways, including VEGF signaling and microRNA networks.

### *KRT7* carried by CFPAC-1 cell-derived EVs promotes angiogenesis in PAAD through the VEGF/VEGFR signaling pathway

**Figure 7:**
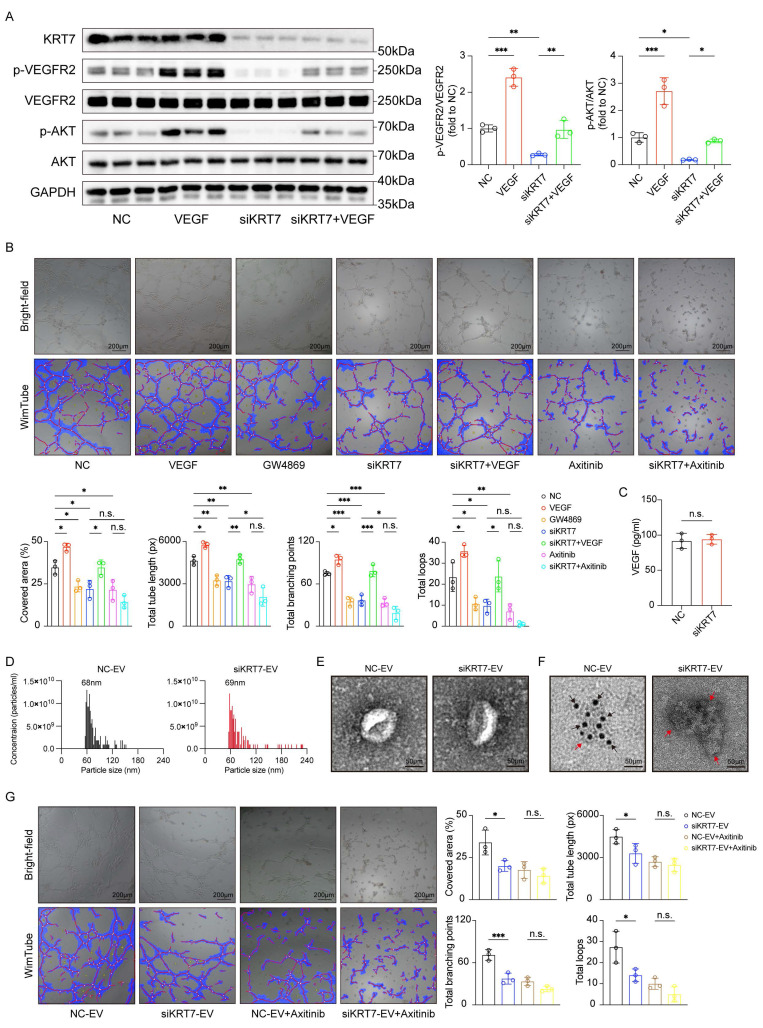
***KRT7* promotes angiogenesis in PAAD through VEGF/VEGFR signaling pathway.** (**A**) Western blotting analysis of protein levels of VEGF signaling pathway components in CFPAC-1 cells transfected with siKRT7 or NC, with or without VEGF treatment. Quantification of p-VEGFR2/VEGFR2 and p-AKT/AKT. (**B**) Representative images of endothelial tube-formation assays under different conditions. Bright-field images (top) and WimTube-analyzed images (bottom) are shown. Quantification of tube-formation parameters, including covered area, total tube lengths, total number of branching points, and total number of loops. (**C**) ELISA analysis for VEGF in CFPAC-1 cells. (**D**) Size distribution of EVs measured by Resistive Pulse Sensing (RPS). Average particle sizes of 68nm for EVs derived from CFPAC-1 cells transfected with NC (NC-EV, left) and 69nm for EVs derived from CFPAC-1 cells transfected with siKRT7 (siKRT7-EV, right) via RPS. (**E**) Representative TEM images of EVs isolated from NC-EV (left), and siKRT7-EV (right). Scale bar, 50 nm. (**F**) Immunoelectron microscopy analysis of *KRT7* in NC-EV (left) and siKRT7-EV (right). Scale bar, 50 nm. Red and black arrows indicate the EVs and *KRT7* carried by EVs after incubation with the anti-*KRT7* primary antibody and the corresponding species-specific colloidal gold conjugate, respectively. (**G**) Representative images of endothelial tube-formation assays following treatment with different EVs, with or without Axitinib. Bright-field images (top) and WimTube-analyzed images (bottom) are shown. Quantification of tube-formation parameters, including covered area, total tube lengths, total number of branching points, and total number of loops. Data are expressed as mean ± SD from three independent experiments. ****p ≤ 0.0001; ***p ≤ 0.001; **p ≤ 0.01; *p ≤ 0.05; n.s., non-significant.

Based on KEGG enrichment results that highlighted the VEGF signaling pathway as a potential mediator, we further examined whether *KRT7* regulates angiogenesis through VEGF/VEGFR signaling using functional and mechanistic analyses in a *KRT7*-knockdown model. Western blot analysis showed that *KRT7* knockdown significantly decreased the phosphorylation of VEGFR2 and AKT, two key components of the VEGF/VEGFR pathway. VEGF treatment partially restored the phosphorylation levels of both VEGFR2 and AKT, suggesting that *KRT7*-driven angiogenesis is dependent on VEGF/VEGFR signaling (**Figure 7A**).

Furthermore, endothelial tube-formation assays in HUVECs showed that conditioned media from *KRT7*-knockdown cells significantly impaired tube formation, as evidenced by reductions in covered area, total tube length, total number of branching points, and total number of loops (**Figure 7B**). The values of these angiogenesis-related parameters partially recovered after VEGF treatment, whereas Axitinib, a VEGFR inhibitor, completely abolished the rescue effect. Similar inhibitory effects were observed following GW4869 treatment, indicating that EV secretion plays a role in the pro-angiogenic activity. When conditioned media from *KRT7*-knockdown cells were combined with Axitinib, no significant difference in angiogenesis was observed compared to using either *KRT7*-knockdown media or Axitinib alone. To assess whether *KRT7* regulates VEGF secretion, VEGF levels in the culture supernatant were quantified by ELISA. No significant difference was observed between the NC and siKRT7 groups, indicating that *KRT7* knockdown in CFPAC-1 cells does not affect VEGF secretion (**Figure 7C**). EVs derived from CFPAC-1 cells were then isolated and characterized. RPS analysis revealed that EVs from both NC and *KRT7*-knockdown cells exhibited similar size distributions, with a peak diameter of approximately 68-69 nm (**Figure 7D**). Transmission electron microscopy (TEM) further confirmed the characteristic vesicular morphology of the isolated EVs (**Figure 7E**). Moreover, immunoelectron microscopy showed that *KRT7* was present in EVs derived from NC cells, whereas EVs from *KRT7*-knockdown cells lacked detectable *KRT7* (**Figure 7F**).

To further evaluate the role of EV-associated *KRT7* in angiogenesis, purified EVs were directly applied to endothelial cells in tube formation assays. Compared with NC-derived EVs, siKRT7-derived EVs significantly suppressed endothelial tube formation, as evidenced by reduced covered area, total tube length, number of branching points, and total loops. Axitinib diminished the pro-angiogenic effect of NC-derived EVs, and no significant differences were observed between the NC-EV + Axitinib and siKRT7-EV + Axitinib groups across all measured parameters (**Figure 7G**). These results indicate that EVs derived from CFPAC-1 cells carrying *KRT7* promote angiogenesis through the VEGF/VEGFR signaling pathway.

## Discussion

In recent decades, survival outcomes for patients with PAAD have remained poor. Despite its high lethality, PAAD has historically received less attention than other malignancies, resulting in relatively limited funding and research efforts. This imbalance has led to its characterization as a “medical emergency” and represents a substantial global burden 18. Several studies have attempted to improve prognostic assessment. For example, Tomonari Asano and colleagues used the Charlson Age-Comorbidity Index (CACI) to predict outcomes in patients undergoing resection for PAAD 19. Similarly, Si Shi *et al*. examined the relationship between prognosis and the strain ratio (SR) measured by endoscopic ultrasonography elastography 20. Lincheng Li and co-workers further demonstrated that CKS1B regulates cancer cell viability and invasion by modulating PD-L1 expression 21. Although these approaches have improved prognostic prediction, a deeper understanding of molecular mechanisms remains essential for developing effective therapies. These findings may help stimulate further interest and investment in improving outcomes for patients with PAAD.

EVs circulate widely within the body and carry diverse molecular cargo. Their lipid bilayer structure protects enclosed components from degradation, making EVs a reliable source of information reflecting the current pathological state. The persistently poor survival of cancer patients, particularly in PAAD, is largely driven by recurrence. Alterations in small metabolites often accompany tumor progression; however, current detection methods are insufficient for the early identification of recurrence risk 22. In many cases, recurrence is detected only after metastasis, limiting opportunities for timely intervention. Advances in detection strategies could extend the window for postoperative management. Bruno Costa-Silva *et al*. reported that PAAD-derived EVs contribute to the formation of a premetastatic niche in the liver 23. At the same time, Vincent Bernard *et al*. showed that longitudinal monitoring of exoDNA and ctDNA through liquid biopsy provides prognostic value 24. Unlike conventional approaches that analyze molecules within complex biological matrices such as blood, circulating EVs can deliver more specific information about target genes, offering substantial utility for both clinical and research applications. Similarly, J. Castillo *et al*. characterized the surfaceome of PAAD-derived exosomes, enabling more precise isolation of tumor-specific material 25. These advances highlight the potential of EV-based approaches to support personalized treatment strategies and provide a strong foundation for future large-scale prospective studies.

*KRT7* has emerged as an important regulator of cancer progression and metastasis through multiple mechanisms across different tumor types. It has been shown to promote invasion and metastasis in colorectal and breast cancers by modulating key signaling pathways and post-transcriptional processes 26,27. In PAAD, *KRT7* is closely associated with metastatic potential, poor prognosis, and regulation of the immune microenvironment, and it has been identified as a key biomarker for survival prediction, particularly through its involvement in immune-related pathways and interactions with tumor-infiltrating immune cells 28. Moreover, *KRT7* has been linked to cell-in-cell structures, suggesting a role in unique cellular interactions that contribute to PAAD progression 29. These findings underscore the multifaceted role of *KRT7* in PAAD and support its potential as both a diagnostic biomarker and a therapeutic target.

Although PAAD is relatively hypovascular compared with other solid tumors, its limited vasculature and angiogenesis-related signaling within the dense stromal microenvironment remain key drivers of tumor progression and poor clinical outcomes. Chongbiao Huang *et al*. demonstrated the significant involvement of the BICC1/LCN2 signaling axis in PAAD angiogenesis 30. Furthermore, PAAD-derived EVs play a pivotal role in promoting angiogenesis. Kai Chen *et al*. reported that exosomal miR-30b-5p, secreted by hypoxic pancreatic cancer cells, promotes endothelial tube formation by targeting and suppressing GJA1, thus driving angiogenesis 31. Targeting bioactive molecules in PAAD-derived EVs has been shown to mitigate angiogenesis. Xufan Cai *et al*. reported that exosomal miR-204 facilitates vasculogenic mimicry (VM) by the maintenance of DVL3-associated stemness, while Ginsenoside Rg3 can significantly reduce angiogenesis and VM by suppressing miR-204 32. These findings indicate that PAAD-associated angiogenesis is sustained by multiple mechanisms, particularly those involving EVs, suggesting the potential to target angiogenesis to improve clinical outcomes in patients.

In this study, by analyzing PAAD and EV-related datasets, nine EV survival-related genes were identified. Functional clustering analysis revealed their prognostic properties, and five prognostic genes were selected to construct a nomogram model that is highly robust for clinical prognosis prediction. The predictive power of immune status was also evaluated considering the dense fibrous stroma 33 and extensive immunosuppression 34 characteristic of the PAAD microenvironment. One limitation of this study is that internal resampling was not conducted during model development, and the proportional hazards assumption underlying the Cox models was not formally evaluated. Future studies should incorporate resampling-based internal validation and perform proportional hazards diagnostics to enhance the robustness and interpretability of the Cox-based prognostic model.

The findings align closely with those from the first systematic review and meta-analysis by Stefania Bunduc *et al*. 35. Moreover, *KRT7* was selected as a target gene for experimental validation in a clinical cohort and *in vitro*. The results demonstrated that *KRT7* knockdown inhibited the oncological behavior of the CFPAC-1 PAAD cell line. RNA-seq suggested that *KRT7* may promote tumor angiogenesis, metabolic adaptation, and immune evasion in PAAD by altering cytoskeletal integrity and polarity and regulating key cancer-related signaling pathways, including the VEGF signaling pathway and microRNA networks. EV isolation and endothelial tube formation assays further confirmed that *KRT7* is a key driver of angiogenesis in PAAD via the VEGF/VEGFR signaling pathway. Further studies are needed to determine whether targeting EV-associated *KRT7* can offer translational therapeutic benefits, potentially by reducing pro-tumorigenic signaling within the tumor microenvironment.

This innovative work on PAAD prognosis demonstrates the potential of using liquid biopsy methods with EVs to predict tumor progression and metastasis. The constructed prognostic model provides a valuable tool for clinicians to tailor treatments based on individual RS. Furthermore, using an in-house cohort, *in vitro* assays, RNA-seq, and tube-formation assays, this study is the first to propose that *KRT7* promotes PAAD angiogenesis *via* the VEGF/VEGFR signaling pathway. These findings provide a novel perspective for future research on EV-targeted anti-angiogenic therapies for PAAD.

## Supplementary Material

Supplementary figures and tables.

## Data Availability

The datasets analyzed during the current study are available in ExoRbase (http://www.exorbase.org/), UCSC Xena (https://xenabrowser.net), GEO database (GSE62452_GPL6244, GSE78229_GPL6244, and GSE111672 https://www.ncbi.nlm.nih.gov/geo), and HPA dataset (https://www.proteinatlas.org). RNA-seq data are deposited on the National Center for Biotechnology Information (NCBI); accession numbers GSE283773 (https://www.ncbi.nlm.nih.gov/geo/query/acc.cgi?acc=GSE283773).

## Abbreviations

PAAD: Pancreatic adenocarcinoma
EVs: Extracellular vesicles
UVA: Univariate Cox regression analysis
LASSO: Least absolute shrinkage and selection operator
RNA-seq: RNA sequencing
miRNAs: microRNAs
EMT: Epithelial-mesenchymal transition
OS: Overall survival
DEGs: Differentially expressed genes
FC: Fold change
GO: Gene ontology
MFs: Molecular functions
CCs: Cellular components
BPs: Biological processes
KEGG: Kyoto Encyclopedia of Genes and Genomes
SNV: Single nucleotide variation
CNV: Copy number variation
RS: Risk score
HRG: High-risk group
LRG: Low-risk group
ROC: Receiver operating characteristic
MVA: Multivariate Cox regression analysis
TIME: Tumor immune microenvironment
ssGSEA: single-sample gene set enrichment analysis
DCs: Dendritic cells
NK: Natural killer
CR: Complete response
PR: Partial response
SD: Stable disease
PD: Progressive disease
IMDM: Iscove's Modified Dulbecco's Medium
FBS: Fetal bovine serum
P/S: Penicillin-streptomycin
DMEM: Dulbecco's Modified Eagle's Medium
IHC: Immunohistochemistry
NC: Negative control siRNA
KM: Kaplan‒Meier
HR: Hazard ratio
AUC: Area under the curve
IPF: Independent prognostic factor
DCA: Decision curve analysis
HPA: Human Protein Atlas
CACI: Charlson age comorbidity index
SR: Strain ratio
VM: Vasculogenic mimicry
