# Supplementary material for: From Extracellular Vesicles-Related Genes to Angiogenesis: KRT7 as a Modulator of the VEGF/VEGFR signaling-dependent angiogenesis in Pancreatic Adenocarcinoma
Source: Int J Med Sci. 2026 May 1;23(6):2054–71. doi: 10.7150/ijms.129654 (PMC13181378; doi:10.7150/ijms.129654)
Supplement: Supplementary file 1 — Supplementary figures and tables. [file ijmsv23p2054s1.pdf]

Supplementary Materials for

From Extracellular Vesicles-Related Genes to Angiogenesis: KRT7 as a Modulator of the VEGF/VEGFR signaling–dependent angiogenesis in Pancreatic Adenocarcinoma

Tianyin Ma *et al.*

*Corresponding author. Email: wangqikun131@163.com (Q.W.);

dr.med.mingtian@whu.edu.cn (M.T.)

This file includes:

Figure S1 to S2

Table S1 to S7

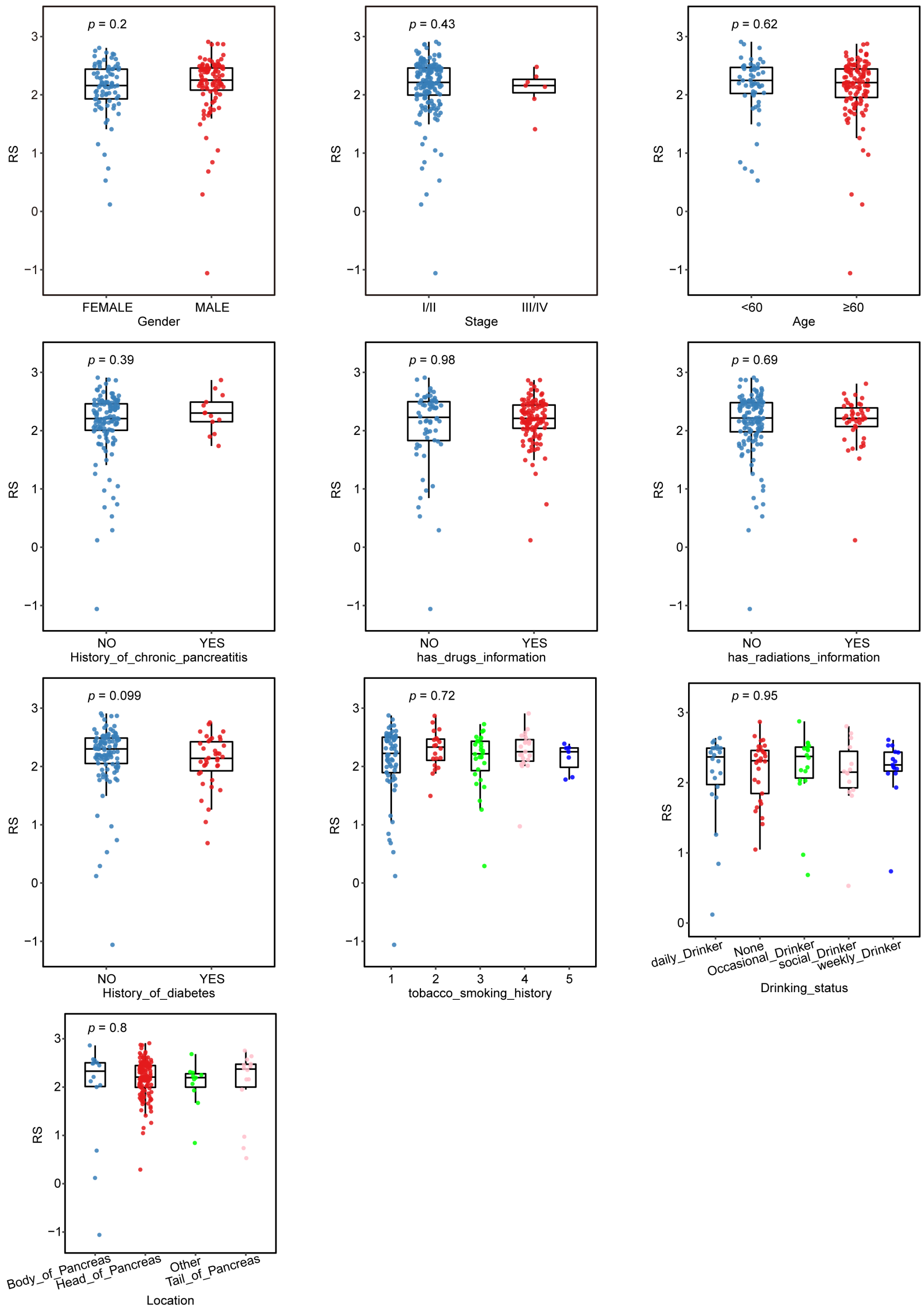
p = 0.2
RS
FEMALE
MALE
Gender
p = 0.43
I/II
III/IV
Stage
p = 0.62
<60
≥60
Age
p = 0.39
NO
YES
History_of_chronic_pancreatitis
p = 0.98
has_drugs_information
p = 0.69
has_radiations_information
p = 0.099
History_of_diabetes
p = 0.72
1
2
3
4
5
tobacco_smoking_history
p = 0.95
daily_Drinker
None
Occasional_Drinker
social_Drinker
weekly_Drinker
Drinking_status
p = 0.8
Body_of_Pancreas
Head_of_Pancreas
Other
Tail_of_Pancreas
Location

Figure S1: Boxplots showing the association between RS and clinical features

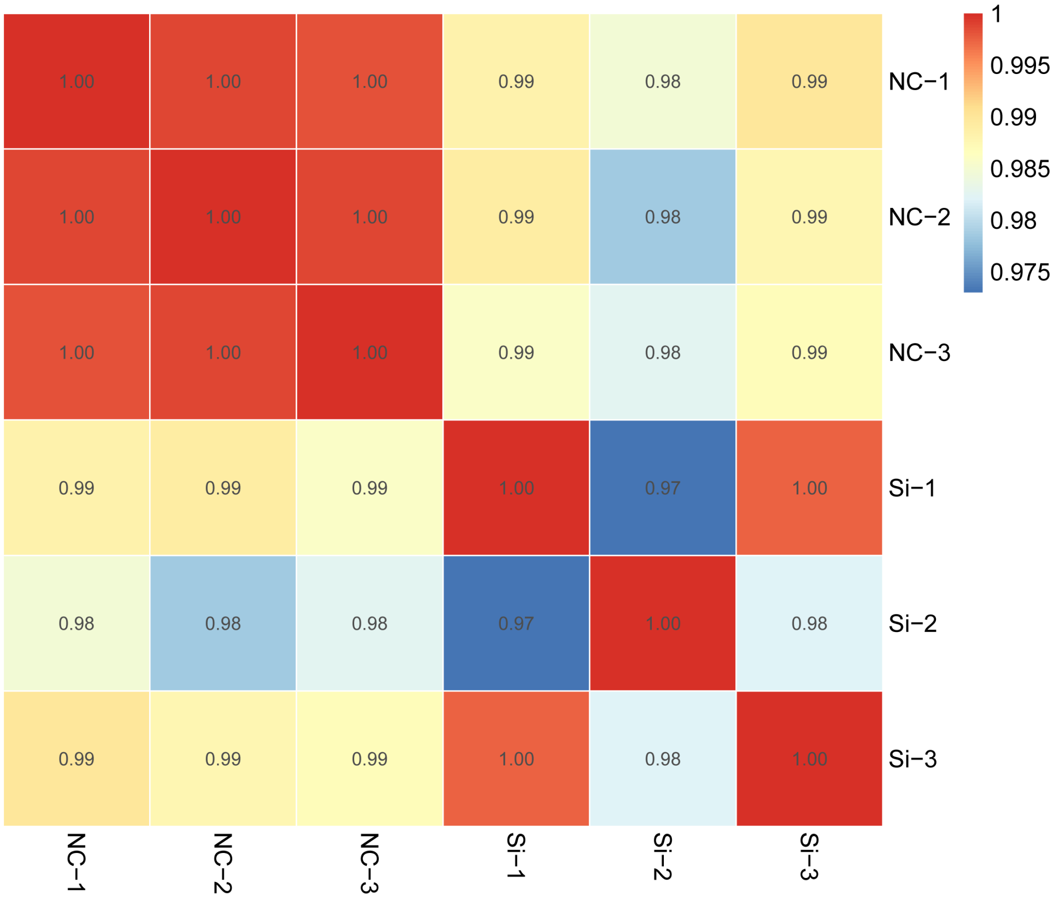

1.00
0.99
0.98
0.97
NC−1
NC−2
NC−3
Si−1
Si−2
Si−3
1
0.995
0.99
0.985
0.98
0.975

Figure S2. Correlation analysis.

Table S1. Clinical information table for the TCGA-PAAD training set.

Characteristics	N	%
Age		
< 60	55	31
≥ 60	122	69
Stage		
Stage I/II	167	94.3
Stage III/IV	7	4.0
Missing	3	1.7
Race		
ASIAN	11	6.4
BLACK OR AFRICAN AMERICAN	6	3.5
WHITE	155	90.1
Gender		
Female	80	45.2
Male	97	54.8
Histological type		
Pancreas-Adenocarcinoma Ductal Type	146	83.4
Pancreas-Adenocarcinoma-Other Subtype	25	14.3
Pancreas-Colloid (mucinous non-cystic) Carcinoma	4	2.3
Grade		
G1/G2	125	70.6
G3/G4	50	28.2
Missing	2	1.1
Vital status		
Alive	85	48

Dead	92	52
KRAS_Mutation		
No	41	23.1
Yes	129	72.9
Missing	7	4
TP53_Mutation		
No	62	35
Yes	108	61
Missing	7	4
Dringing_status		
Daily_Drrinker	22	12.4
Weekly_Drinker	15	8.5
Social_Drinker	14	7.9
Occasional_Drinker	18	10.2
None	29	16.4
Missing	79	44.6
Tobacco_smoking_history		
1	66	37.3
2	20	11.3
3	28	15.8
4	23	13.0
5	7	4.0
Missing	33	18.6
History_of_diabetes		

No	108	61.0
Yes	38	21.5
Missing	31	17.5
History_of_chronic_pancreatitis		
No	128	72.3
Yes	13	7.4
Missing	36	20.3
Location		
Head_of_Pancreas	138	78.0
Body_of_Pancreas	14	7.9
Tail_of_Pancreas	11	7.9
Other	11	6.2
Has_radiations_information		
No	134	75.7
Yes	43	24.3
Has_drugs_information		
No	60	33.9
Yes	117	66.1

Table S2. Clinical information table for the GSE62452_GPL6244 validation set.

Characteristics	N	%
Stage		
Stage I/II	48	75.00
Stage III/IV	16	25.00
Grade		
G1/G2	34	53.13
G3/G4	30	46.87
RS		
Low	33	50.77
High	32	49.23

Table S3. Clinical information table for the GSE78229_GPL6244 validation set.

Characteristics	N	%
Stage		
Stage I	4	8.16
Stage II	45	91.84
Grade		
G1/G2	26	54.17
G3/G4	22	45.83
RS		
Low	25	51.02
High	24	48.98

Table S4. Sample information statistics.

Dataset	Tumor samples	Normal samples
PAAD_ExoRbase	164	118
TcgaTargetGtex-PAAD	178	171
GSE62452_GPL6244	65	0
GSE78229_GPL6244	49	0

Table S5. Clinical data for in-house data.

Characteristics	N	%
Age		
≤ 60	17	51.5
≥ 60	16	48.5
Sex		
Female	12	36.4
Male	21	63.6
Smoking		
Never	24	72.7
Ever	9	27.3
Drinking		
Never	31	93.9
Ever	2	6.1
Histologic differentiation		
Low	10	30.3
Moderate and high	23	69.7
Tumor stage		
T1+T2	25	75.8
T3+T4	8	24.2
Nodal stage		
N0	5	15.2
N1-2	28	84.8
Metastatic stage		
MX/M0	32	97.0
M1	1	3.0

Table S6. All primer and siRNA sequences.

Name	Sequence (5'-3')
KRT7-F	CGGCATCATCGCTGAGGTCAA
KRT7-R	GCCTGGAGGGTCTCAAACTTGG
GAPDH-F	ACCACAGTCCATGCCATCAC
GAPDH-R	TCCACCACCCTGTTGCTGTA
siNC-F	UUCUCCGAACGUGUCACGU
siNC-R	ACGUGACACGUUCGGAGAA
siKRT7#1-F	CCGUGAAUAUCUCUGUGAUGA
siKRT7#1-R	UCAUCACAGAGAUAUUCACGG
siKRT7#2-F	CCUGAAUGAUGAGAUCAACUU
siKRT7#2-R	AAGUUGAUCUCAUCAUUCAGG

Table S7. Antibody information.

Name	Supplier	Catalog Number
Cytokeratin 7-specific Polyclonal antibody	proteintech	17513-1-AP
HRP-conjugated Goat Anti-Rabbit IgG(H+L)	proteintech	SA00001-2
HRP-conjugated GAPDH Mouse mAb	ABclonal	AC035
Pan-Akt Rabbit mAb	ABclonal	A22412
Phospho-Akt-S473 Rabbit mAb	ABclonal	AP1208
VEGFR2/CD309 Rabbit mAb	ABclonal	A25502
Phospho-VEGF Receptor 2-Y1175 Rabbit pAb	ABclonal	AP0382
10 nm gold-conjugated anti-rabbit secondary antibody	Solarbio	K1034G-G35
